# Clinical significance of gelsolin-like actin-capping protein expression in oral carcinogenesis: an immunohistochemical study of premalignant and malignant lesions of the oral cavity

**DOI:** 10.1186/1471-2407-8-39

**Published:** 2008-02-01

**Authors:** Hitomi Nomura, Katsuhiro Uzawa, Takashi Ishigami, Yukinao Kouzu, Hirofumi Koike, Katsunori Ogawara, Masashi Siiba, Hiroki Bukawa, Hidetaka Yokoe, Hitoshi Kubosawa, Hideki Tanzawa

**Affiliations:** 1 Department of Clinical Molecular Biology, Graduate School of Medicine, Chiba University, 1-8-1 Inohana, Chuo-ku, Chiba, 260-8670, Japan; 2 Division of Dentistry and Oral-Maxillofacial Surgery, Chiba University Hospital, 1-8-1 Inohana, Chuo-ku, Chiba, 260-8670, Japan; 3 Department of Pathology, Chiba Municipal Aoba Hospital, 1273-2 Aoba-cho, Chuo-ku, Chiba 260-0852, Japan; 4 Center of Excellence (COE) Program in the 21st Century, Graduate School of Medicine, Chiba University, 1-8-1 Inohana, Chuo-ku, Chiba, 260-8670, Japan

## Abstract

**Background:**

Gelsolin-like actin-capping protein (CapG) is a ubiquitous gelsolin-family actin-modulating protein involved in cell signalling, receptor-mediated membrane ruffling, phagocytosis, and motility. CapG has generated great interest due to its oncogenic function in the control of cell migration or invasion in a variety of cancer cells. We previously applied proteomic methods to characterize differentially expressed proteins in oral squamous-cell carcinoma (OSCC) cells and detected significantly high expression levels of CapG in OSCC-derived cell lines compared to human normal oral keratinocytes. In the current study, to further determine the potential involvement of CapG in OSCC, we evaluated the status of CapG protein and mRNA expression in human oral premalignant lesions (OPLs) and primary OSCCs and correlated the results with clinicopathologic variables.

**Methods:**

Matched normal and tumour tissue sections of 79 human primary OSCCs and 28 OPLs were analyzed for CapG expression by immunohistochemistry (IHC). Correlations between CapG-immunohistochemical staining scores of OSCCs and clinicopathologic features were evaluated by Fisher's exact test. Real-time quantitative reverse transcriptase-polymerase chain reaction (qRT-PCR) was used to estimate CapG expression at the mRNA level.

**Results:**

In IHC, substantial up-regulation of CapG protein was observed in primary OSCCs (52%) and OPLs (64%), whereas corresponding normal tissues showed consistently weak or absent immunoreactivity of CapG. qRT-PCR data were consistent with the protein expression status. Moreover, CapG expression was correlated with the TNM stage grading of OSCCs.

**Conclusion:**

Our finding of frequent dysregulated expression of CapG in premalignant and malignant lesions together with an association with an advanced clinical disease stage suggests that CapG could contribute to cancer development and progression and that CapG may have potential as a biomarker and a therapeutic target for OSCC.

## Background

Oral squamous cell carcinoma (OSCC) is a major cause of morbidity and mortality globally, accounting for 275,000 new cases and more than 120,000 deaths annually [1-3]. Despite therapeutic and diagnostic advances, patients often are diagnosed at advanced stages and mortality rates are still increasing [4]. This highlights the need for continued efforts to discover suitable biomarkers for early disease diagnosis and to understand the disease pathogenesis as a first step toward improving treatment. Considering these problems, it is imperative to study oral carcinomas at the genetic level and to characterize the genetic changes responsible for carcinogenesis and tumour behaviour.

Because proteomics-based profiling uniquely allows delineation of global changes in expression patterns resulting from transcriptional and posttranscriptional control and posttranslational modifications, proteomic tolls are used increasingly in the post-genomic era to discover new cancer biomarkers [5,6]. We recently developed a strategy of using proteomics technologies to search for significant molecular biomarkers characteristic of oral carcinogenesis [7-10]. Among the proteins identified, CapG expression was found to be up-regulated in OSCC-derived cell lines compared to human normal oral keratinocytes using a fluorescent two-dimensional differential in-gel electrophoresis (2-D-DIGE) system and matrix-assisted laser desorption/ionization time-of-flight mass spectrometry (MALDI-TOF/MS).

CapG, also known as macrophage capping protein, is a 348-amino acid protein that is ubiquitously expressed in normal tissues and particularly abundant in macrophages [11-13]. CapG is a member of the actin-binding protein, which is crucial for the organization of the actin cytoskeleton. The actin cytoskeleton underlies many cellular functions including the maintenance and mutability of cell shape, motility, adherence, and growth regulation [14-16]. Since these cellular functions have escaped normal control mechanisms during carcinogenesis, the behaviour of actin and actin-binding proteins has undergone intense scrutiny as a potential contributor to malignant transformation and a target for anticancer drug development [17-19]. Evidence indicates that CapG also possesses an oncogenic function involved in the control of cell migration or invasion, and we hypothesized that the protein has potential as an emerging therapeutic target of interest for the treatment of oral cancer. However, it is unclear whether CapG is associated with oral carcinogenesis. The purpose of the current study was to determine CapG protein/mRNA expression in a series of human primary OSCCs and human oral premalignant lesions(OPLs) and correlate the protein expression with the clinical relevance in patients with OSCC.

## Methods

### Tissue specimens

Seventy-nine pairs of primary OSCC samples and corresponding normal oral epithelium tissues or 28 OPLs (diagnosed as oral leukoplakias) were obtained at the time of surgery performed at Chiba University Hospital between 1998 and 2006. All patients provided informed consent according to the protocol that was reviewed and approved by the institutional review board of Chiba University before any procedures were performed. The resected tissues were divided into two parts: one was frozen immediately after removal of the surrounding normal tissue and stored at -80°C until RNA extraction, and another was fixed in 10% buffered formaldehyde solution for pathologic diagnosis and immunohistochemical staining. Histopathologic diagnosis of each tumour specimen was carried out according to the International Histological Classification of Tumors by the Department of Pathology, Chiba University Hospital. Clinicopathologic staging was determined by the TNM classification of the International Union against Cancer. All OSCC samples were histologically confirmed and checked to ensure the presence of tumour in greater than 80% of specimens.

### Cell culture

The OSCC-derived cell lines used in this study were HSC-2, HSC-3, and Ca9-22 (Human Science Research Resources Bank, Osaka, Japan). All OSCC-derived cell lines were cultured in Dulbecco's modified Eagle medium F-12 HAM (Sigma-Aldrich Co.), supplied with 10% heat-inactivated fetal bovine serum (Sigma) and 50 U/ml^-1 ^penicillin and streptomycin (Sigma), and incubated at 37°C in a humidified atmosphere with 5% CO_2_.

### Protein and mRNA extraction

Protein was extracted when the cells reached 80% to 90% confluence; they were washed twice with phosphate buffered saline (PBS), scraped into a tube, and centrifuged briefly. The cell pellets were incubated for 30 min in a lysis buffer containing 7 M urea, 2 M thiourea, 4% w/v CHAPS, and 10 mM Tris pH 8.0, and lysed by sonication (3 × 10 sec pulses on ice). The sample was centrifuged at 13,000 rpm for 20 min. The supernatant containing the cell proteins then was recovered and the protein concentration was measured with a Protein Assay Kit (Bio-Rad Laboratories) and adjusted to 1 mg/ml with lysis buffer. The pH of the protein sample was adjusted to 8.5 with 30 mM Tris-HCl. Total RNA was extracted using Trizol Reagent (Invitrogen Life Technologies) according to the manufacturer's instructions. Each extracted RNA or protein was stored separately at -80°C until use.

### Immunohistochemistry

To examine the cellular distribution of CapG protein in oral lesions, we carried out immunohistochemical staining on 4-μm sections of paraffin-embedded specimens. Briefly, after deparaffinization and hydration, the slides were pretreated in 10 mM sodium citrate buffer (pH 6.0) in a microwave oven for 5 min at 95°C. The endogenous peroxidase activity was quenched by 30-min incubation in a mixture of 0.3% hydrogen peroxide solution in 100% methanol. After being washed with PBS buffer, the sections then were incubated with primary antibody affinity-purified goat antihuman CAPG polyclonal antibody (1:500 dilution; Santa Cruz Biotechnology, Santa Cruz, CA; catalogue number: sc-33084) at room temperature in a moist chamber overnight. After being washed with PBS buffer, the slides were treated with biotinylated secondary antibody for 1 hr followed by colour development in 3, 3'-diaminobenzidine tetrahydrochloride (DAKO JAPAN Inc., Kyoto, Japan). Finally, the slides were lightly counterstained with hematoxylin. A negative control was established by replacing the primary antibody with PBS. To quantitate the state of CapG protein expression, the mean percentage of positive tumour cells was determined in at least five random fields at 400 × magnification in each section. The intensity of the CapG immunoreaction was scored as follows: 1+, weak; 2+, moderate; and 3+, intense. Three target cell types, i.e., normal, premalignant, and malignant epithelial cells, were identified for scoring. The percentage of CapG-positive cells and the staining intensity then were multiplied to establish a CapG-immunohistochemical (CapG-IHC) staining score. Cases with a CapG-IHC score exceeding 78.68 (maximum score of normal tissues) were considered positive. Two independent pathologists, neither of whom had knowledge of the patients' clinical status, made these judgments.

### Western blot analysis

To confirm the specificity of the CapG antibody used, three Western blot examinations were carried out on three OSCC-derived cell lines (HSC-2, HSC-3, and Ca9-22). Protein extracts were electrophoresed on 11% sodium dodecyl sulfate-polyacrylamide gel electrophoresis gels, transferred to polyvinylidene difluoride (PVDF) membranes (Bio-Rad), and blocked for 1 hr at room temperature in 5% skim milk. Immunoblot PVDF membranes were washed five times with 0.1% Tween 20 in TBS (TBS-T), and 2 μg ml-1 affinity-purified goat antihuman CAPG polyclonal antibody (1:5000 dilution; Santa Cruz Biotechnology) was added directly to the TBS-T solution for 2 hr at room temperature. The PVDF membranes were washed again and incubated with 1:10,000 ratio of anti-goat IgG (Nichirei Biosciences Inc., Tokyo, Japan; catalogue number: 414161) as a secondary antibody for 20 min at room temperature. The membranes then were incubated with Enhanced ChemiLuminescence (ECL)+ -HRP HORSERADISH PEROXIDASE? substrate solution included in the ECL+ kit (Amersham Biosciences), and immunoblotting was visualized by exposing the membrane to Hyperfilm (Amersham Biosciences).

### mRNA expression analysis

The expression levels of *CapG *mRNA were examined in 50 OSCC specimens from patients with primary tumours among the OSCC cases studied by IHC staining. Control reactions were prepared in parallel without reverse transcriptase (RT). Before cDNA synthesis, residual genomic DNA was removed from the total RNA using DNase I treatment (DNA-free; Ambion, Austin, TX). The primer sequences used to analyze *CapG *mRNA expression were 5-CTCACAGCTGACAAGGCAAA-3 (nucleotides 965–984) and 5-CCACCCTCATTTCCAGTCC-3 (nucleotides 1303–1321). The sequences of specific primers were checked before use to avoid amplification of genomic DNA or pseudogenes by the Primer3 program [20]. Amplified products were analyzed by 3% agarose gel electrophoresis to ascertain size and purity. Real-time quantitative RT-polymerase chain reaction (qRT-PCR) was performed with a single method using the LightCycler FastStart DNA Master SYBR Green I kit (Roche, Mannheim, Germany). To prepare the standard curve, 3 μg of total RNA from normal oral tissue was reverse-transcribed with Superscript RT (Life Technologies, Grand Island, NY) and oligo-d(T)12–18 primer, after which serial dilutions were made corresponding to cDNA transcribed from 300, 30, 3.0, and 0.3 ng of total RNA. PCRs using LightCycler apparatus were carried out in a final volume of 20 μl of reaction mixture consisting of 2 μl of FirstStart DNA Master SYBR Green I mix, 3 mM MgCl_2_, and 0.2 μl of primers, according to the manufacturer's instructions. The reaction mixture then was loaded into glass capillary tubes and submitted to an initial denaturation at 95°C for 10 min, followed by 45 rounds of amplification at 95°C (10 sec) for denaturation, 58°C (10 sec) for annealing, and 72°C for extension, with a temperature slope of 20°C/sec, performed in the LightCycler. The transcript amount for *CapG *was estimated from the respective standard curves and normalized to the *GAPDH *transcript amount determined in corresponding samples.

### Statistical analysis

Differences in gene expression levels between CapG-positive and CapG-negative cases were calculated with the Mann-Whitney's *U*-test. Correlations between CapG-IHC scores and clinicopathologic features were evaluated by Fisher's exact test. The criterion for statistical significance was P < 0.05. Mean values are shown with the standard error.

## Results

### Immunohistochemical analysis

**Figure 1:**
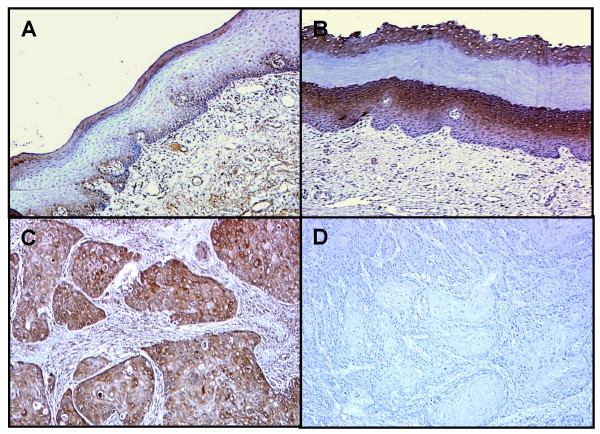
**Representative results of immunohistochemical staining of CapG in normal tissues, OPLs, and primary OSCCs**. A: Normal oral tissue exhibits negative CapG protein expression (original magnification × 100); B: CapG-positive case of OPL. The immunoreaction is enhanced in the spinous layer (original magnification × 100); C: CapG-positive case of OSCC (stage IV). Strong positive immunoreactivity for CapG is detected in the nuclear and cytoplasm (original magnification × 200); D: The same OSCC sample is immunostained in the absence of the primary antibody (original magnification × 200).

IHC staining was performed using a series of surgical specimens, including 79 OSCCs with corresponding normal tissues and 28 OPLs. Representative results for CapG protein expression in normal oral tissues, OPLs, and primary OSCCs are shown in Figure 1. Normal oral mucosal specimens had no or faint CapG immunoreactivity in almost all epithelium cells and were considered CapG-negative. In contrast, 41 of 79 (52%) cases of OSCCs examined had significantly increased expression of CapG immunoreactivity in the nucleus and cytoplasm of the tumour cells (IHC score > 78.68; maximum score of normal tissues). In OPL specimens, positive CapG immunoreactivity was detected distinctly on spinous cells in 18 of 28 cases (64%). Stromal components such as fibroblasts, lymphocytes, endothelial cells, and macrophages also exhibited positive CapG immunoreactivity, the last of which CapG is known to be homogeneously expressed in both normal and tumour specimens. In the current study, we focused on CapG expression in epithelium cells. The specificity of the primary antibody also was evaluated. A single band was recognized at the appropriate molecular weight of CapG protein on Western blot analysis as reported by Johnston *et al *[21] (see additional file 1). Furthermore, no IHC staining was observed when OSCC tissue was analyzed without the primary antibody (Figure 1D).

**Figure 2:**
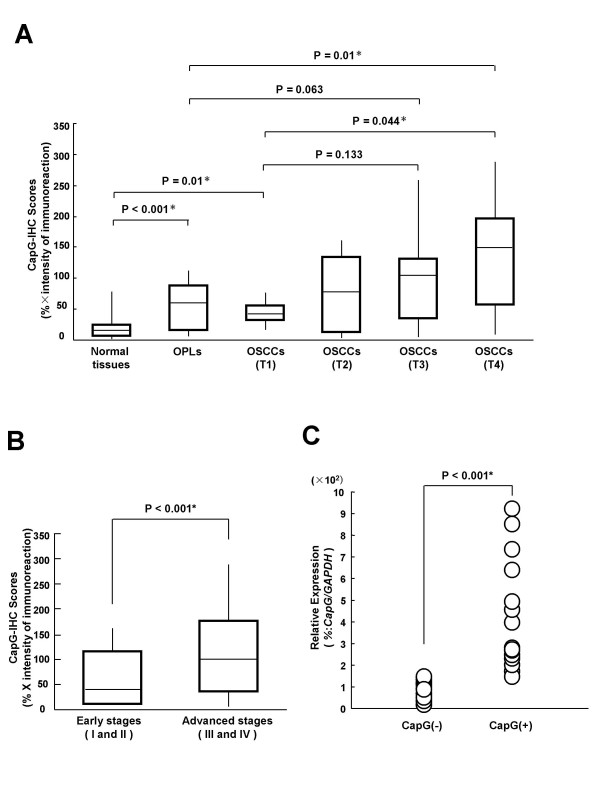
**CapG protein and mRNA expression in normal tissues, OPLs, and primary OSCCs.** A: CapG protein expression in OPLs (n = 28) and OSCCs (n = 79) is significantly higher than in normal oral tissues (n = 79; P < 0.01, Mann-Whitney's U-test). The results are expressed as the mean ± SD; B: States of CapG protein expression in early-stage (I and II) OSCC (n = 27) and advanced stages (III and IV) OSCC (n = 52) are compared. CapG protein expression in advanced stages is significantly higher than in the early stages (P < 0.001, Mann-Whitney U-test); C: Comparison of CapG mRNA expression levels between CapG-positive and CapG-negative cases in primary OSCCs classified by IHC analysis. There is a significant difference in the CapG mRNA expression levels between the negative and positive cases (P < 0.001, Mann-Whitney's U-test).

The CapG-IHC scores for normal tissues, OPLs, and OSCCs ranged from 0 to 79 (mean, 17.14), 6 to 204 (mean, 65.36), and 3 to 287 (mean, 96.19), respectively. CapG expression levels in primary OSCCs and OPLs were significantly (P < 0.01) higher than those in normal oral tissues (Figure 2A). In contrast, there was no significant (P = 0.80) difference in CapG-IHC scores between OSCCs and OPLs. The correlation between the clinicopathologic characteristics of the patients with OSCC and the status of CapG expression is summarized in Table 1. CapG protein expression was correlated with OSCC tumor size (P = 0.014). Moreover, the CapG-IHC scores for early stages (I and II) and advanc *ed s*tages (III and IV) ranged from 3.62 to 160.3 (mean, 60.54) and 4.95 to 287.4 (mean, 107.4), respectively. The CapG expression levels were significantly higher in the OSCC group with advanced-stage disease compared with the group with early-stage disease (Mann-Whitney U-test, P < 0.001) (Figure 2B).

**Table 1:** Correlation between the Expression of CapG and Clinical Classification in OSCCs.

		**Immunostaining Results No. patients (%)**		
			
**Clinical Classification**	**Total**	**Capg (-)**	**Capg (+)**	**P Value**^**a**^
Age at surgery (years)				
< 60	30	15 (50)	15 (50)	
60 =<, < 70	22	11 (50)	11 (50)	0.920408
70 =<	27	12 (44)	15 (56)	
Gender				
Male	53	23 (43)	30 (57)	0.337982
Female	26	15 (58)	11 (42)	
T-primary tumour				
T1	4	4 (100)	0 (0)	
T2	25	16 (64)	9 (36)	0.0140021
T3	24	10 (42)	14 (58)	
T4	26	8 (31)	18 (69)	
N-regional lymph node				
N (-)	50	27 (54)	23 (46)	0.242807
N (+)	29	11 (38)	18 (62)	
Stage				
I	4	4 (100)	0 (0)	
II	15	10 (67)	5 (33)	0.0191771
III	19	10 (53)	9 (47)	
IV	41	14 (34)	27 (66)	
Histopathologic type				
Well differentiated	46	23 (50)	23 (50)	
Moderately differentiated	28	12 (43)	16 (57)	0.714701
Poorly differentiated	5	3 (60)	2 (40)	
Tumour site				
Tongue	34	20 (59)	14 (41)	
Gingiva	26	9 (35)	17 (65)	0.110879
Oral floor	8	5 (63)	3 (37)	
Buccal mucosa	6	1 (17)	5 (83)	
Oropharynx	4	3 (75)	1 (25)	
Lip	1	0 (0)	1 (100)	

Leukoplakia	28	10 (36)	18 (64)	

^a^P < 0.05 was considered significant.

### mRNA expression analysis

qRT-PCR analysis data were matched to protein expression levels studied by IHC scores. The CapG mRNA expression levels significantly increased in primary tumours of randomly selected CapG-positive cases (n = 10) compared with randomly selected CapG-negative cases (n = 10, Mann-Whitney U-test, P < 0.001) (Figure 2C). The relative mRNA expression levels in negative and positive cases ranged from 0.16 to 1.15 (mean, 0.75) and 2.72 to 9.22 (mean, 5.67), respectively. Therefore, CapG mRNA expression levels were consistent with the protein expression.

## Discussion

Tumour-associated processes such as invasion and metastasis are critically dependent on dynamic alterations in the organization of the actin cytoskeleton. Dysregulation of actin-based motility is a prominent factor in cell transformation and probably is associated with carcinogenesis [22,23]. To date, a large number of actin-binding proteins have been cloned, many of which are involved in the malignant transformation process; and distinctive protein expression patterns of some of these genes in cancer and progressive carcinogenesis processes have been observed [24-28]. Previously, we reported significant overexpression of the actin-binding protein CapG expression at the protein level in OSCC-derived cell lines using a 2-D-DIGE system and MALDI-TOF/MS [7].

CapG is a member of the gelsolin superfamily of the actin-binding proteins [29]. In addition to respective roles in actin filament remodelling, the proteins of the gelsolin superfamily have specific roles in several cellular processes, including cell motility, signal control, and apoptosis and regulation of phagocytosis [30,31]. Variations in expression of the gelsolin superfamily proteins are thought to affect major cytoskeletal changes during differentiation and carcinogenesis, and considerable evidence has shown a significant association between the proteins and a wide range of human malignancies including OSCCs [32-35]. Experimental evidence has shown that CapG also is crucial for regulating cell motility [36,37]; however, its exact function in the development and progression of malignant tumours remains controversial. Watari et al. reported an apparent decrease in CapG protein levels in some human tumor cell lines compared with their corresponding benign counterparts. In addition, those investigators showed that CapG protein expression reduces the ability of a transformed cell to induce tumour formation, suggesting that CapG is a tumour suppressor gene [38]. However, accumulating evidence has indicated a number of theories on the possible function of CapG as a tumor activator. A recent study identified CapG as a target of the AP-1 transcription factor complex, which has emerged as a critical regulator of gene expression in response to the activation of a variety of oncogenic signal transduction cascades, including c-Fos and c-Jun [39]. De Corte et al. reported that overexpression of CapG promotes cancer cells to invade collagen through the Ras-phosphoinositide 3-kinase signaling pathway [40]. More recently, they reported that targeting human cancer cells, including breast and prostate cancer cells with an RNAi procedure against CapG, significantly reduced the invasive and motile properties of both cells examined as well as cell aggregation [41]. Similar results were obtained by others in pancreatic cancer cells [42]. These findings support the hypothesis that CapG also is a tumour activator. Furthermore, significant overexpression of CapG was reported at both the mRNA and protein levels in several types of human primary tumours, i.e., ocular melanomas [43], glioblastomas [44], and pancreatic ductal adenocarcinomas [42], all of which are aggressive cancers that kill patients by metastasis and local invasion. Because OSCC is also a solid neoplasm exhibiting aggressive tumour phenotypes, we hypothesized that CapG is a potential emerging therapeutic target of interest for the treatment of oral cancer. However, the status of CapG in OSCC remains unclear and therefore we selected it for further investigation.

To confirm our hypothesis, we determined the protein/mRNA expression in a series of human primary OSCCs using IHC. By evaluating the CapG-IHC scores, significant up-regulation was evident in the primary OSCCs compared with normal tissues (P < 0.01). The CapG protein expression levels in primary OSCCs were significantly associated with tumor size (P = 0.014). In pancreatic ductal adenocarcinomas, a clinical study also showed that high CapG expression was correlated with increased tumour size [42], which was consistent with results obtained in the current study. Moreover, the state of CapG protein expression differed significantly between the early stages (I and II) and the advanced stages (III and IV) (Mann-Whitney U-test, P < 0.001). Given the known roles of CapG in cytoskeletal organization and cell migration, these findings indicated the potential clinical significance of CapG as a marker or prospective therapeutic target for the most aggressive forms of OSCCs. Furthermore, high levels of CapG protein expression were detected even in the OPLs examined, suggesting that CapG may play an important role in early-stage OSCC development. However, the development of OSCC is generally predicated on the development of multiple clonal genetic alterations [45,46], and additional research is needed to establish whether and how CapG-stained OPLs give rise to strongly CapG-stained OSCCs.

## Conclusion

Significant overexpression of the actin-binding protein CapG was detected not only in 52% of OSCCs but also in 64% of OPLs. Enhanced expression of CapG was associated with large tumour size and advanced staging of OSCCs. Based on our data, we concluded that CapG may initiate or activate the neoplastic process of OSCC cells including the regulation of biologic behaviour of aggressive forms of cells rather than as a suppressor. In addition, CapG may serve as a useful diagnostic biomarker or a therapeutic target to identify and treat patients with advanced OSCCs. The current results together with previously reported evidence suggest that CapG provides an attractive molecular target for decreasing invasiveness and the metastatic properties of tumors. Further studies may provide insights into the process of tumorigenicity and for planning new treatment strategies.

## Abbreviations

**CapG**, gelsolin-like actin capping protein; **OSCC**, oral squamous-cell carcinoma; **OPL**, human oral premalignant lesion;**IHC**, immunohistochemistry; **qRT-PCR**, real-time quantitative reverse transcriptase-polymerase chain reaction; **2-D-DIGE**, two-dimensional differential in-gel electrophoresis; **MALDI-TOF/MS**, matrix-assisted laser desorption/ionization time-of-flight mass spectrometry

## Competing interests

The author(s) declare that they have no competing interests.

## Authors' contributions

HN carried out and coordinated the study, conducted IHC and data analysis, and drafted the manuscript. KU participated in the study design, interpretation of data, and revision of the manuscript. TI, HK, and YK performed and evaluated the immunohistochemical analysis. KO isolated RNAs and performed qRT-PCR analysis. MS contributed to and supervised the statistical analysis. HB and HY were involved in the sample acquisition, sample selection, clinical data acquisition, and preparing the manuscript. HK did the pathology review. HT supervised and controlled the entire study. All authors reviewed and commented on successive drafts of the manuscript and approved the final manuscript.

## Pre-publication history

The pre-publication history for this paper can be accessed here:



## Supplementary Material

Additional file 1 Control experiments for CapG primary antibody used in IHC. Western blot examination of CapG protein in three OSCC-derived cell lines (HSC-2, HSC-3, and Ca9-22) using the primary antibody used in IHC. All OSCC-derived cell line extracts exhibit a single band for CapG protein expression. Click here for file
