# Supplementary figures and images for: Clinical significance of gelsolin-like actin-capping protein expression in oral carcinogenesis: an immunohistochemical study of premalignant and malignant lesions of the oral cavity
Source: BMC Cancer. 2008 Feb 1;8:39. doi: 10.1186/1471-2407-8-39 (PMC2263057; doi:10.1186/1471-2407-8-39)

## Slide 1
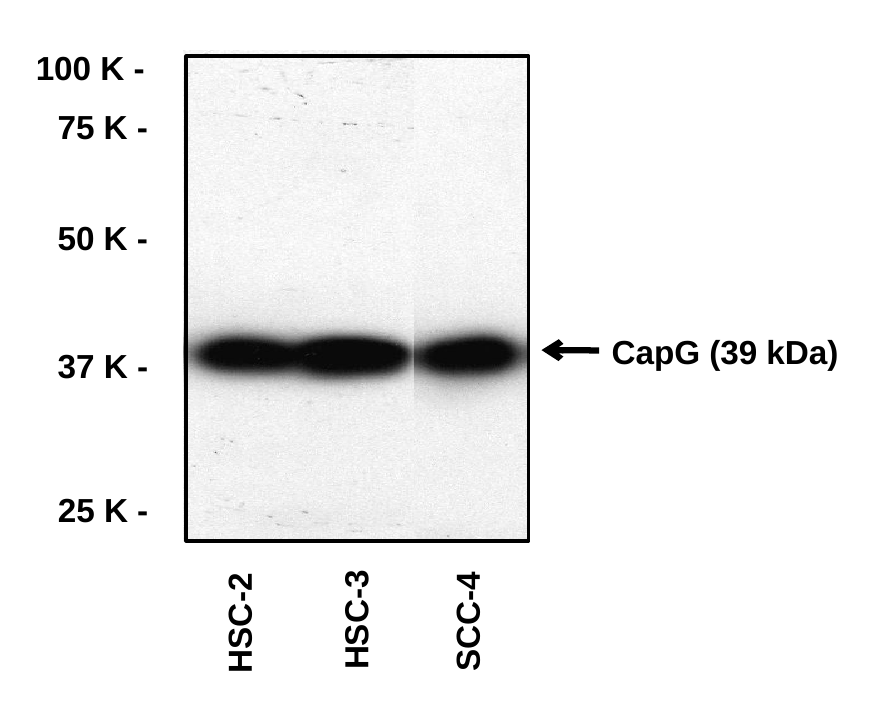

100 K -
75 K -
50 K -
37 K -
25 K -
HSC-3
HSC-2
SCC-4
CapG (39 kDa)

Supplement: Additional file 1 — Control experiments for CapG primary antibody used in IHC. Western blot examination of CapG protein in three OSCC-derived cell lines (HSC-2, HSC-3, and Ca9-22) using the primary antibody used in IHC. All OSCC-derived cell line extracts exhibit a single band for CapG protein expression. [file 1471-2407-8-39-S1.ppt]
